# Exosomes and exosomal miRNAs: A new therapy for intervertebral disc degeneration

**DOI:** 10.3389/fphar.2022.992476

**Published:** 2022-09-08

**Authors:** Zhichao Li, Yan Wu, Guoqing Tan, Zhanwang Xu, Haipeng Xue

**Affiliations:** ^1^ First College of Clinical Medicine, Shandong University of Traditional Chinese Medicine, Jinan, China; ^2^ Department of Orthopedics, The First Affiliated Hospital of Shandong First Medcial Unversity, Jinan, China; ^3^ Department of Orthopedics, Affiliated Hospital of Shandong University of Traditional Chinese Medicine, Jinan, China

**Keywords:** exosome, intervertebral disc degeneration, microRNA, exosomal miRNA, regenerative medicine

## Abstract

Low back pain has been found as a major cause of global disease burden and disability. Intervertebral disc degeneration is recognized as the vital factor causing low back pain. Intervertebral disc degeneration has a complex mechanism and cannot be avoided. Traditional strategies for the treatment of intervertebral disc degeneration cannot meet the needs of intervertebral disc regeneration, so novel treatment methods are urgently required. Exosomes refer to extracellular vesicles that can be released by most cells, and play major roles in intercellular material transport and information transmission. MicroRNAs have been identified as essential components in exosomes, which can be selectively ingested by exosomes and delivered to receptor cells for the regulation of the physiological activities and functions of receptor cells. Existing studies have progressively focused on the role of exosomes and exosomal microRNAs in the treatment of intervertebral disc degeneration. The focus on this paper is placed on the changes of microenvironment during intervertebral disc degeneration and the biogenesis and mechanism of action of exosomes and exosomal microRNAs. The research results and deficiencies of exosomes and exosomal microRNAs in the regulation of apoptosis, extracellular matrix homeostasis, inflammatory response, oxidative stress, and angiogenesis in intervertebral disc degeneration are primarily investigated. The aim of this paper is to identify the latest research results, potential applications and challenges of this emerging treatment strategy.

## Introduction

The entire spine of the human body has been damaged from birth by exercise or other causes. Over time, the damage has evolved into low back pain. Almost everyone has a short history of acute low back pain in their lifetime. However, some people will develop chronic low back pain and suffer from pain continuously (Vlaeyen et al., 2018). Low back pain has been found as the leading cause of disability worldwide (GBD 2015 Disease and Injury Incidence and Prevalence Collaborators, 2016). It can be attributed to various known or unknown diseases. Although low back pain has complex causes, intervertebral disc (IVD) degeneration (IVDD) is undoubtedly a vital factor. IVDD has become a social hot issue with the exponential increase in the incidence rate (Hartvigsen et al., 2018; Yang et al., 2020).

Traditional treatment strategies include surgical and non-surgical treatments, capable of achieving limited intervention in the IVD microenvironment through nerve root decompression, removal of degenerated IVD, pharmacological or non-pharmacological therapy, or interventional therapy. However, traditional treatment strategies focusing on mitigating symptoms do not facilitate the regeneration of IVD at a cellular level, and they even accelerate the degeneration of adjacent stages (Harper and Klineberg, 2019; Harmon et al., 2020). Accordingly, novel therapeutic strategies are strategies restoring the physiological structure and biomechanical function of IVD to treat the disease, instead of IVD regeneration strategies that can mitigate symptoms (Binch et al., 2021). The above strategies are basically assigned to cell-based therapy and cell-free therapy. To be specific, cell therapy, a bioactive scheme of IVDD, has aroused wide attention (DiStefano et al., 2022). However, all the evidence has demonstrated that degenerated IVD tissue is in a “harsh” environment that is characterized by high osmolarity, high mechanical stress, hypoxia, nutritional deficiencies, as well as increased levels of acidity and inflammation (Dowdell et al., 2017; Urban, 2002; Liang et al., 2018). Thus, all relevant therapies (e.g., cell therapy) aim to improve this microenvironment. As a matter of fact, cell therapy has placed an emphasis on this harsh microenvironment at the early stage. Although cell therapy shows some functional improvements as compared with untreated controls, no further breakthrough has been made thus far (Wuertz et al., 2008; Sakai and Andersson, 2015). Besides, there are a series of problems (e.g., cell selection, delivery, viability and phenotypic stability, as well as treatment time, regulatory problems, and high cost) (Sakai and Andersson, 2015; Meisel et al., 2019; Croft et al., 2021). Accordingly, although cell therapy remains promising for IVDD, cell-free therapy as an alternative may achieve similar or greater therapeutic benefits (Burdick et al., 2013; DiStefano et al., 2022). Moreover, exosomes produced by cells and containing bioactive molecules (e.g., effective cytokines, growth factors, and miRNAs) play a vital role in treating numerous diseases (e.g., nervous system diseases, cardiovascular diseases, respiratory diseases, hepatic diseases, and renal diseases) (Yin et al., 2022; Williams et al., 2019; de Freitas et al., 2021; Guo H et al., 2021; Zhou L et al., 2022; Blijdorp et al., 2022). An increasing number of researchers have begun to investigate the therapeutic role of exosomes in IVDD. In this paper, the focus is placed on exosomes and important miRNAs in exosomes, and their latest research results, mechanism of action, potential applications and challenges in IVDD are summarized.

## Intervertebral disc and intervertebral disc degeneration

### Anatomy and function of intervertebral disc

IVD, the largest avascular tissue in the human body, is composed of fibrocartilaginous tissue that can be divided into three distinct parts, including a highly hydrated gelatinous nucleus pulposus (NP) located at the center, annulus fibrosis (AF) composed of collagen fiber lamellas around NP, as well as cartilaginous endplate (CEP) with hyaline cartilage structure that connects the vertebral bodies. The above complex structure takes on a critical significance to transmit and absorb the mechanical load between vertebral bodies and maintain flexibility (Dowdell et al., 2017).

NP is largely occupied by notochordal cells (NCs) at the early stage of growth and development. Subsequently, chondrocyte-like cells exhibiting poor water retention capacity tend to replace resident NCs. The above age-related transition can result in a reduction in IVD’s hydration status and more stronger and less compliant functional spinal unit overall (Molladavoodi et al., 2020). The extracellular matrix (ECM) of NP primarily consists of proteoglycans and type II collagen. Aggrecan combined with hyaluronic acid has been found to be the most abundant in proteoglycans. Their negatively charged side chains enable NP to retain water and have high osmotic pressure, thus maintaining the height and mechanical elasticity of IVD (Erwin and Hood, 2014). Furthermore, the above high concentrations of aggrecan will result in less innervation and angiogenesis in IVD (García-Cosamalón et al., 2010). Under hypoxic conditions, NP cells (NPCs) initiate a cascade of cellular responses by upregulating hypoxia-inducible factor (HIF), which plays a certain role in glycolysis and mitochondrial energy metabolism to present an acidic environment within IVD (Li et al., 2013).

AF falls into internal and external parts, which include 15–25 layers of highly packed inclined collagen fiber lamellas and interspersed proteoglycans (Matsushima et al., 2021). The lamellas cross each other, and adjacent layers are reversed. These lamellas, connected by translamellar bridges composed of elastin and type VI collagen, exhibit significant mechanical nonlinearity and anisotropy (Tavakoli et al., 2016; Chu et al., 2018; Torre et al., 2019). AF composition and structure exhibit progressive characteristics from the inside to the outside. The outer region primarily consists of type I collagen secreted by nearly 95% of elongated fibroblast-like cells, and the content of type I collagen in the inner region close to the NP is reduced to 5%, while the type II collagen secreted by round fibrocartilage-like cells tends to increase (Torre et al., 2019; Kamali et al., 2021).

**FIGURE 1:**
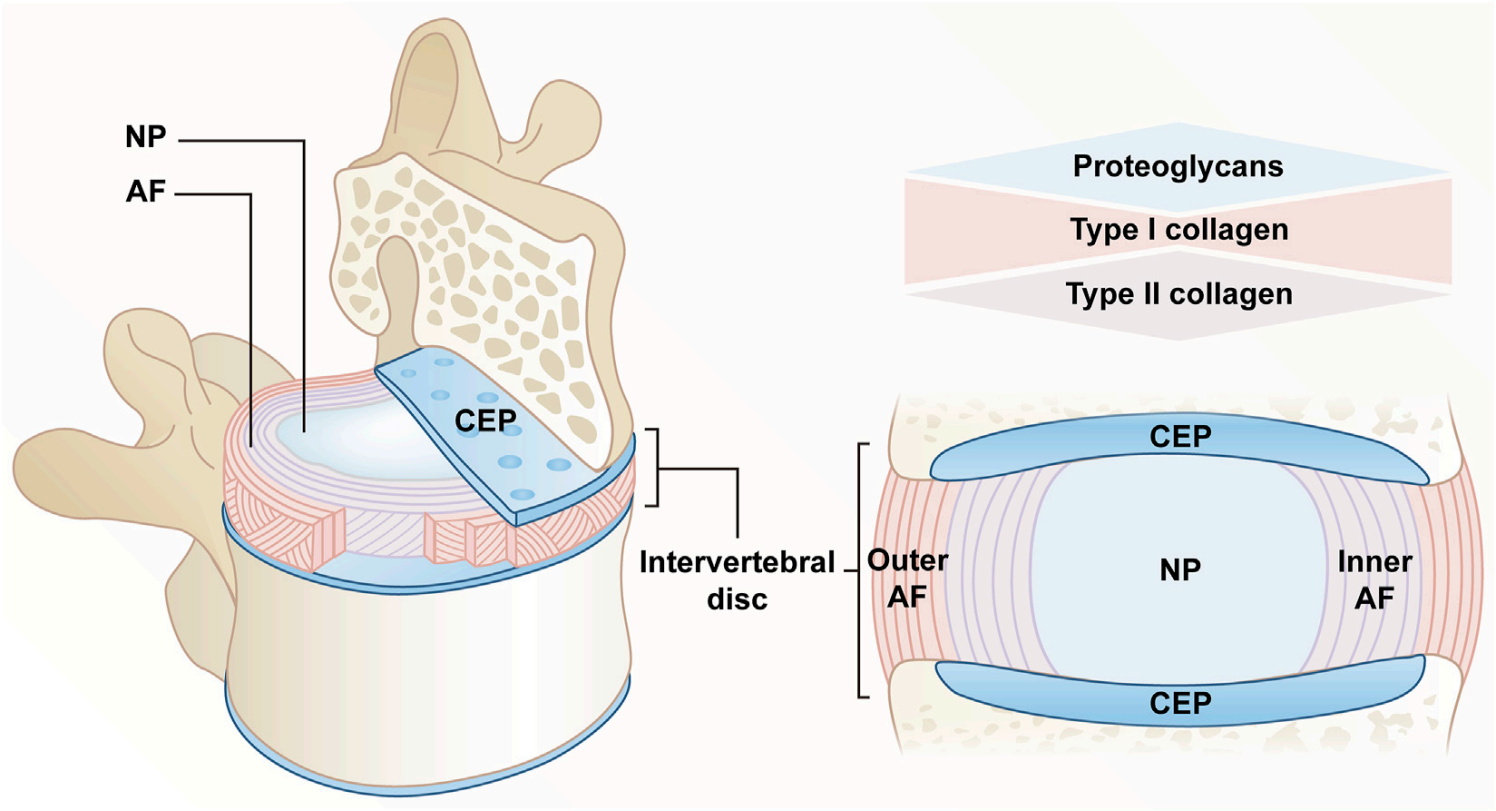
Anatomy of a normal IVD. The IVD consists of a central NP, an AF surrounding the NP, and a CEP connecting the upper and lower cones. The extracellular matrix of NP is mainly composed of proteoglycans and type II collagen. AF is divided into two parts, the content of type I collagen gradually decreases from outside to inside, and type II collagen gradually increases. CEP confines NP and AF within the anatomical boundaries, consisting mainly of proteoglycans and type II collagen.

Similar to other parts of IVD, the main components of CEP consist of water, type II collagen and proteoglycans, which are characterized by structural semipermeable barrier and load-bearing functions (Pattappa et al., 2012). From the structural perspective, CEP is responsible for separating IVD from vertebrae and limiting NP and AF in their anatomical boundaries. As a semipermeable barrier, CEP accounts for the fluid exchange of IVD, as well as the exchange of nutrients and metabolic waste; it also accounts for evenly distributing pressure from IVD to the vertebral body, so as to perform its weight-bearing function (Pattappa et al., 2012; Hodgkinson et al., 2019; Kamali et al., 2021) (Figure 1).

### Pathogenesis of intervertebral disc degeneration

IVDD occurs consistently with age. When NCs in NP are progressively lost in early life, catabolic processes begin to be dominant (Clouet et al., 2009; Risbud et al., 2010). The delicate balance between catabolism and anabolism is lost in IVD, and a series of degradation processes start and are facilitated by numerous factors (e.g., genetic and environmental changes) (Ravichandran et al., 2022). There are some critical pathological changes during IVDD, consisting of abnormal synthesis and degradation of ECM, cellular senescence and death, increased angiogenesis and innervation, aging of AF, as well as calcification of CEP (Ohnishi et al., 2022; Wang et al., 2020).

At first, NCs stimulate the proliferation of NPCs and synthesize proteoglycans, especially due to the secretion of connective tissue growth factor (CTGF). When NCs are gradually lost, and the dialogue with NPS cells ends, IVDD occurs (Erwin et al., 2006). At the early stage of IVDD, the productions of aggrecan and type II collagen are inhibited, the water of hyaluronic acid polymerized into proteoglycan is lost, whereas the productions of type I collagen, fibronectin, and other proteoglycans are facilitated (Ohnishi et al., 2022; Yee et al., 2016). This finding is correlated with an upregulated expression of matrix-degrading proteins (e.g., MMPs and ADAMTS). The former accounts for cleaving fibrillar collagen, while the latter is responsible for degrading aggrecan (Le Maitre et al., 2007). Moreover, their increase exceeds their natural inhibitor, the tissue inhibitor of metalloproteinase (TIMPs) (Le Maitre et al., 2007). The ECM degradation can be further facilitated by cellular senescence and death, and apoptosis is a well-established factor in IVDD that can be induced by several factors (e.g., abnormal mechanical load, oxidative stress, and inflammation) (Ding et al., 2012; Cao et al., 2022). Besides, other common cell death processes (e.g., necroptosis, pyroptosis, ferroptosis, and autophagy) are correlated with IVDD (Ohnishi et al., 2022).

**FIGURE 2:**
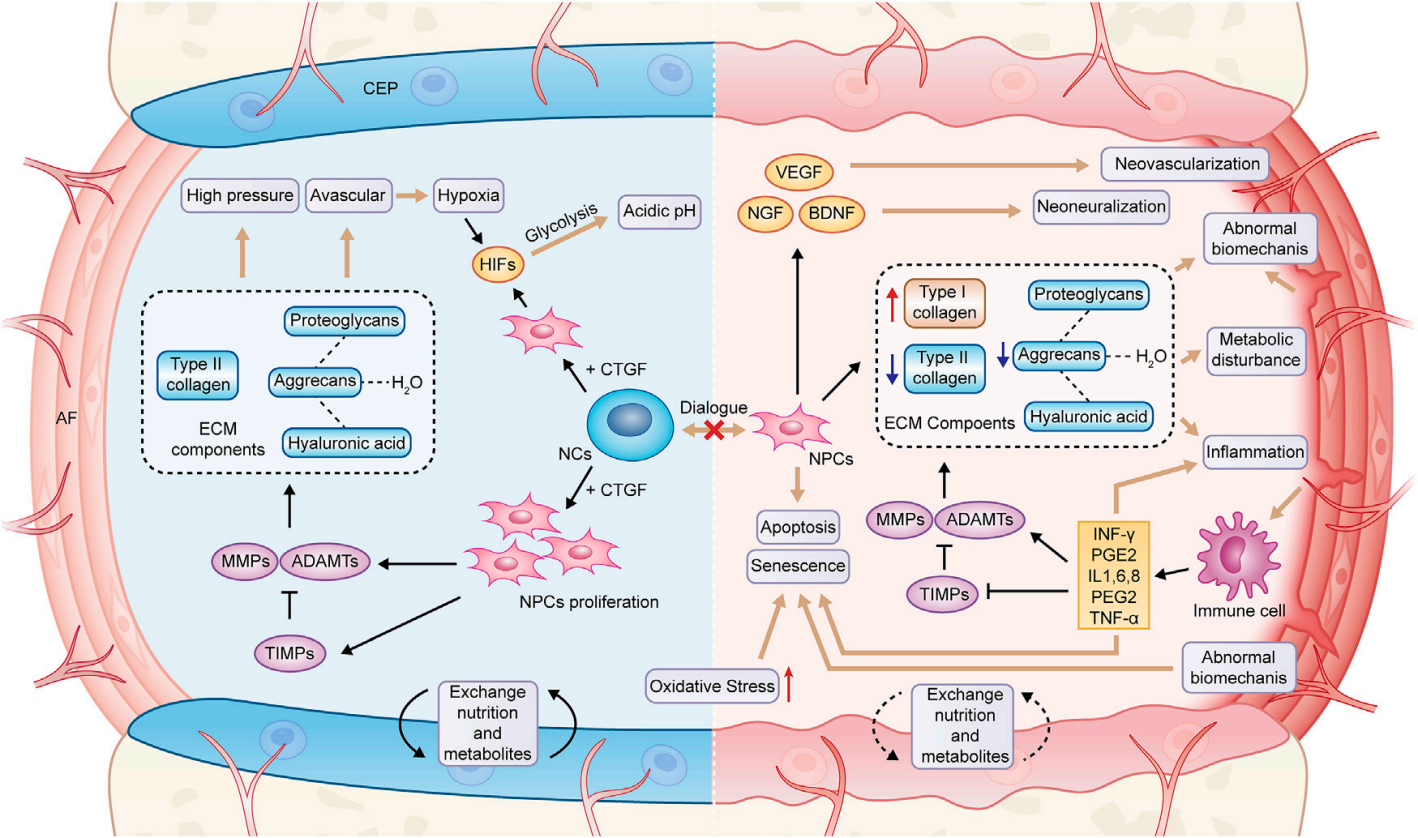
Pathological changes in degenerated IVD. NCs, notochord cells; NPCs, nucleopulpocytes; AF, annulus fibrosus; CEP, cartilage endplate; ECM, extracellular matrix; HIFs, hypoxia-inducible factors; MMPs, metalloproteinases; ADAMTs, metalloproteinase with thrombospondin motifs; TIMPs, tissue inhibitors of metalloproteinases; CTGF, connective tissue growth factor; VEGF, vascular endothelial growth factor; NGF, nerve growth factor; BDNF, brain-derived neurotrophic factor; IL-1,6,8, interleukin-1,6,8; PGE2, prostaglandin E2; TNF-α, tumor necrosis factor; IFN-γ, interferon-gamma.

As a response to tissue damage, inflammation involves a vascular response as well as activation and recruitment of immune cells, however, with IVD being an avascular tissue, it is not surprising that the inflammatory response is different in this case (Molinos et al., 2015). Degradation products after metabolic disorder may be the cause of inflammatory response of IVD (Medzhitov, 2008). It was observed that the expression of inflammatory cytokines secreted by degenerated IVD cells in the IVDD model [e.g., IL-1β, IL-6, IL-8, TNF-α, interferon-gamma (IFN-γ) and prostaglandin E2 (PGE2)] significantly increased (Lyu et al., 2021). Furthermore, the activities of MMPs, ADAMTS and TIMPs are regulated by the above proinflammatory factors, thus remodeling ECM from anabolism to catabolism (Le Maitre et al., 2005). Moreover, the decrease of the content of proteoglycans leads to the disappearance of the barrier that prevents the growth of blood vessels and nerves into NP. As more vascular endothelial growth factor (VEGF), nerve growth factor (NGF) and brain-derived neurotrophic factor (BDNF) are released, blood vessels and nerves grow inward when the natural response of IVD to mechanical load is damaged and microcracks appear (Khan et al., 2017). In addition, when AF is no longer able to maintain NP, the immune system recognizes NP as “non-self” and the immune response is delivered, macrophages, lymphocytes, and other inflammatory cells are recruited and activated to eliminate the foreign body, accompanied by inward growth of angiogenic and neurogenic processes, macrophages secrete TNF-α, IFN-γ, and PGE2, which are key factors in discogenic pain and build an active pro-inflammatory feedback loop with the involvement of activated B and T lymphocytes (Molinos et al., 2015; Sun et al., 2020a). As an important nutritional pathway of IVD, calcification of CEP hinders the exchange of nutrients and metabolites in IVD, which is also an important reason for metabolic disorder of ECM. The above complex microenvironment in degraded IVD will facilitate the production of ROS in IVD cells and lead to the formation of a positive feedback loop (Feng et al., 2017). When the balance of generation and elimination of ROS is disrupted to induce oxidative stress, it further exacerbates degradation and inflammation of ECM and facilitates cellular senescence and apoptosis (Cao et al., 2022) (Figure 2).

### Biological therapy of intervertebral disc degeneration

Traditional surgical procedures including resection and transforaminal lumbar interbody fusion of degenerative IVDs or restricted interventions with conservative therapies are now invariably linked to loss of function, unsatisfactory outcomes, or the accelerated emergence of secondary complications (Harper and Klineberg, 2019; Harmon et al., 2020). Therefore, many biological therapies based on the improvement of the disc microenvironment and structural regeneration have gained attention, such as growth factor therapy, gene therapy, and cell therapy. Some studies have reported the role of various cytokines, including bone morphogenetic protein-7 (BMP-7), BMP-2, transforming growth factor-β (TGF-β), basic fibroblast growth factor (bFGF), epidermal growth factor (EGF), growth and differentiation factor-5 (GDF-5) and insulin-like growth factor-1 (IGF-1), etc. in restoring the height of degenerated IVD, reestablishing ECM metabolic homeostasis and inhibiting apoptosis of degenerated IVD cells (Miyamoto et al., 2006; Ellman et al., 2008; Liang et al., 2010; Cho et al., 2013; Travascio et al., 2014). However, this therapy may be less successful in treating IVDD in its latter phases, and there is debatable about the half-life, vector choice, injection frequency, and safety following repeated injections (Kennon et al., 2018).

Gene therapy introduces genes into target cells *via in vivo* viral or non-viral vectors, and these cells with a controlled genetic makeup will produce proteins that support IVD regeneration and have greater long-term efficacy than injected growth factors (Sampara et al., 2018). However, gene therapy research has mostly remained in the laboratory due to the risk of potential complications associated with viral vectors, uncontrollable gene expression due to erroneous injections, greater adaptation to non-degenerative diseases, and the high cost of research and development (Gonçalves and Paiva, 2017; Sampara et al., 2018).

Among several approved cellular and molecular approaches, stem cell therapy shows great potential in the field of regeneration, as this therapy can restore damaged organs in a variety of debilitating syndromes, including the restoration of the microenvironment and structural regeneration within degenerating IVDs (Fernandez-Moure et al., 2018; Ekram et al., 2021). Numerous investigations, however, have shown that transplanted cells are unable to survive in the hypoxic IVD environment, much less the more intricate and “hostile” milieu of the degenerating disc (Ryu et al., 2020). Therefore, more rational cell selection, delivery and protection modalities are needed to improve the viability and phenotypic stability of transplanted cells (Sakai and Andersson, 2015; Meisel et al., 2019; Croft et al., 2021). Cell-free therapies may bring surprises as new alternative therapies, and exosomes, which can exert the same therapeutic effect as the origin stem cells have become the focus of researchers’ attention (Cosenza et al., 2017). They avoid the risks associated with stem cell transplantation and exhibit tremendous promise in the treatment of IVDD by carrying their cargo, especially miRNAs.

## Exosomes and exosomal miRNAs

### Biogenesis, composition and internalization of exosomes

**FIGURE 3:**
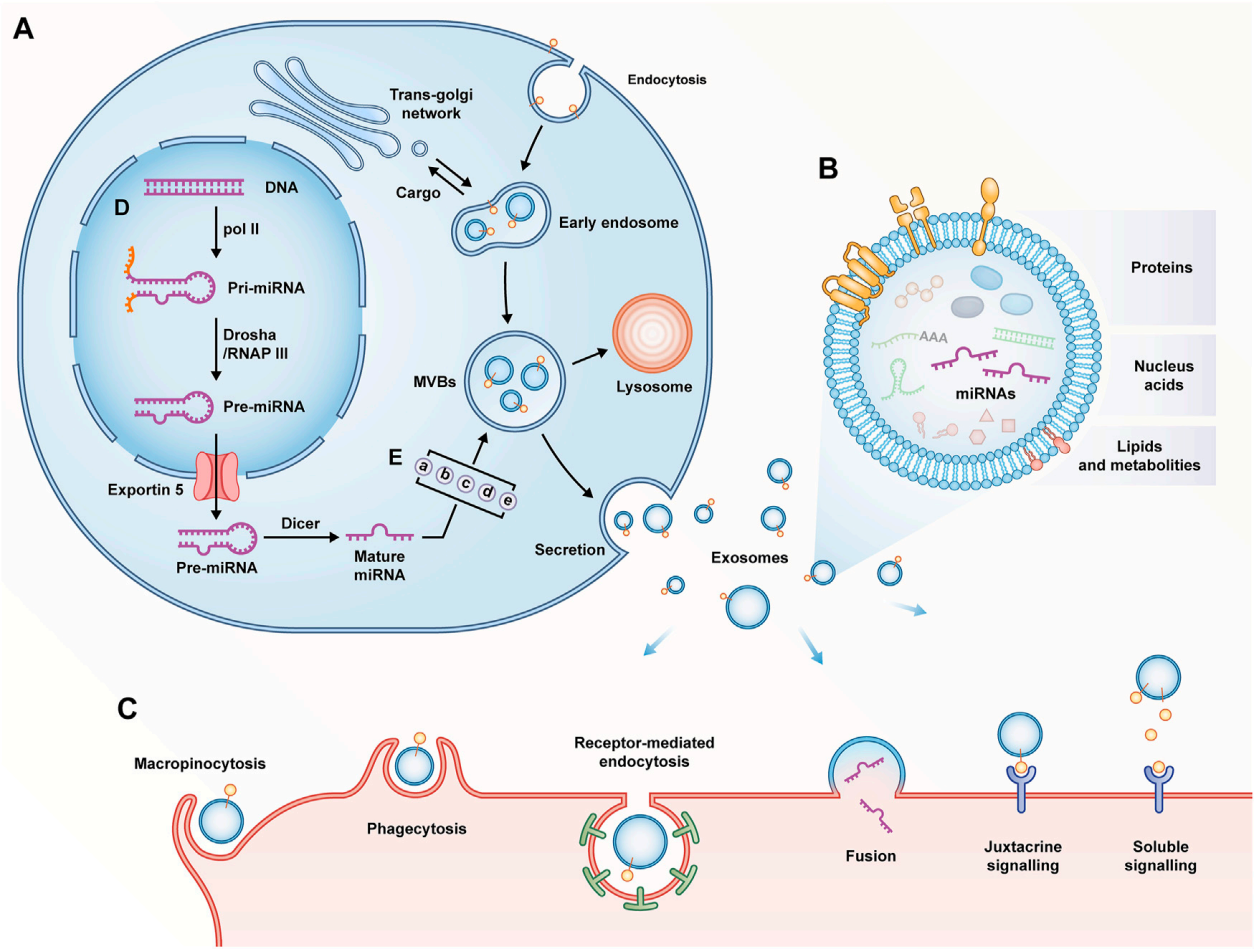
**(A)** Biogenesis of exosomes; **(B)** Composition of exosomes; **(C)** Internalization of exosomes; **(D)** Biogenesis of miRNAs. Pol II, RNA polymerase II; RNAP III, RNA polymerase III; **(E)** Mechanisms of miRNAs classification as exosomes: (a) miRISC-dependent pathway; (b) nMase-2-dependent pathway; (c) miRNA sequence motifs and guide proteins-dependent pathway; (d) 3′miRNA sequence-dependent pathway; (e) cellular availability of miRNAs.

Almost all cell types release extracellular vesicles (EVs), which basically fall into three categories, including apoptotic bodies, ectosomes, and exosomes. Exosomes are different from others since they originate from the endosomal pathway involved in endocytic recycling or lysosomal degradation. This is a strictly and uniquely regulated intracellular process in which composition and function may be already determined when they are secreted into the extracellular space (Kalluri and LeBleu, 2020; Al et al., 2022). The above process consists of several stages, including the formation and development of early endosomes, the formation of intraluminal vesicles (ILV) in MVB, and the transport and fusion of MVB to plasma membrane to complete the release of exosomes (Hessvik and Llorente, 2018). To be specific, the invagination of the plasma membrane is accompanied by cell-surface proteins and soluble proteins, and early endosomes are formed with the participation of the trans-Golgi network and the endoplasmic reticulum (Jan et al., 2019). Subsequently, early endosomes mature into late endosomes through vector selection (Hu et al., 2022). The endosomal sorting complex required for transport (ESCRT) accounting for the transport mechanism can direct the biogenesis of exosomes, and ESCRT-0 recognizes ubiquitinated goods and initiates the pathway. It is transferred to the endosomal membrane with the help of ESCRT I, II and III; with the accumulation of substances, it facilitates the invagination of the endosome to form multiple ILVs, i.e., the future exosomes. At this time, the endosome is also termed MVB (Henne et al., 2013; Yáñez-Mó et al., 2015). Moreover, ESCRT is also regulated by accessory proteins TSG101, ALIX, and VPS4 (Trajkovic et al., 2008). Furthermore, exosome biogenesis can proceed in an ESCRT-independent mechanism (Stuffers et al., 2009). Lastly, MVB can serve as a transporter to deliver contents into lysosomes for degradation; it can also fuse with the cytoplasmic membrane under the regulation of Rab family proteins to release luminal vesicles, namely exosomes (Ostrowski et al., 2010; van Niel et al., 2018) (Figure 3A).

No matter how the biogenesis of exosomes proceeds, they are composed of phospholipid bilayer structures that encapsulate lipids, proteins, and nucleic acids (Kumari and Anji, 2022). Lipids, an essential part of exosome membrane, include cholesterol, sphingyelin, ceramide, etc. Although exosomes contain specific proteins of the cell of origin, there are also fixed collections of proteins unrelated to the cell of origin, which include tetraspanins (CD63, CD81, CD9), MVB-related proteins (ALIX, TSG101), Flotillin-1, heat shock proteins (Hsp60, Hsp70, Hsp90), Rab family proteins, metabolic enzymes, etc. (Pegtel and Gould, 2019). Furthermore, exosomes are enriched with abundant nucleic acids including DNA, mRNAs, and non-coding RNAs (e.g., miRNAs, lncRNAs, and circRNAs) (Jeppesen et al., 2019) (Figure 3B).

After being expelled, exosomes enter body fluids from the extracellular space and circulate at least for a short time; they eventually reach recipient cells locally or remotely (McKelvey et al., 2015). The interaction between the surface proteins of exosomes and cell receptors helps exosomes complete adhesion. Subsequently, the receptor cells achieve internalization of exosomes via soluble and juxtacrine signaling, fusion, macropinocytosis, receptor/raft-mediated endocytosis, as well as phagocytosis. After internalization is complete, the horizontal gene of the exosome contents is transferred into the cytoplasm of the recipient cell and takes effect (Zhang et al., 2019) (Figure 3C).

### Biogenesis, sorting and function of exosomal miRNAs

In 2007, valadi et al. first systematically described RNAs in exosomes (Valadi et al., 2007). Similar to proteins, some of the above RNAs are specific RNAs of origin cells, while some RNAs are fixed regardless of cellular origin (Gibbings et al., 2009). miRNAs have been found as the most representative members of non-coding RNAs in exosomes, which are nearly 22 nucleotides in length. It degrades or inhibits the expression of mRNAs at the post-transcriptional level by directly binding to recognition motifs in the 3′-untranslated region (UTR) of target mRNAs (Treiber et al., 2019). The biogenesis of miRNAs proceeds via a well-characterized mechanism. In the nucleus, miRNA genes are transcribed by RNA polymerase II to form primary miRNAs (pri-miRNAs). Subsequently, the pri-miRNAs are processed into precursor miRNAs (pre-miRNAs) by the Drosha complex. After the protein exportin 5 translocates the pre-miRNA to the cytoplasm, the pre-miRNA matures to a miRNA under the transformation of the Dicer complex (Bartel, 2004) (Figure 3D). Subsequently, mature miRNAs are sorted into exosomes. As revealed by existing research, the above process involves five mechanisms, including (I) miRNA-induced silencing complex (miRISC)-dependent path way, (II) neutral sphingomyelinase-2 (nMase-2) -dependent pathway; (III) miRNA sequence motifs and guide proteins-dependent pathway; (IV) 3′-miRNA sequence-dependent pathway, as well as (V) cellular availability of miRNAs (Bhome et al., 2018) (Figure 3E). Lastly, miRNAs selected into exosomes are released by the cells of origin and input into the recipient cells to play a certain role. Compared with the origin cells, exocrine contains some more abundant miRNAs with a higher proportion, thus revealing the presence of a selective sorting mechanism for miRNAs in exosomes and highlighting their specific roles in the transfer and post-transcriptional regulation of miRNAs in recipient cells (Nolte-'T et al., 2012). Furthermore addition, the regulatory role of miRNAs on downstream target genes is one-to-many, which constitutes a complex and huge regulatory network. This pleiotropic phenomenon enables miRNAs to exert multifunctional effects in tissue homeostasis, pathophysiology, and therapy (Ha and Kim, 2014; DiStefano et al., 2022). The research of miRNAs over the past few years has been highly active in the field of IVDD. It has been confirmed that miRNAs play a certain role in mechanical biology, ECM degradation, cell loss, and inflammation in IVDD. Pathological changes in IVD are closely related to the imbalance of miRNA and its targets (Cazzanelli and Wuertz-Kozak, 2020).

## Application of exosomes and exosomal miRNAs in intervertebral disc degeneration

**FIGURE 4:**
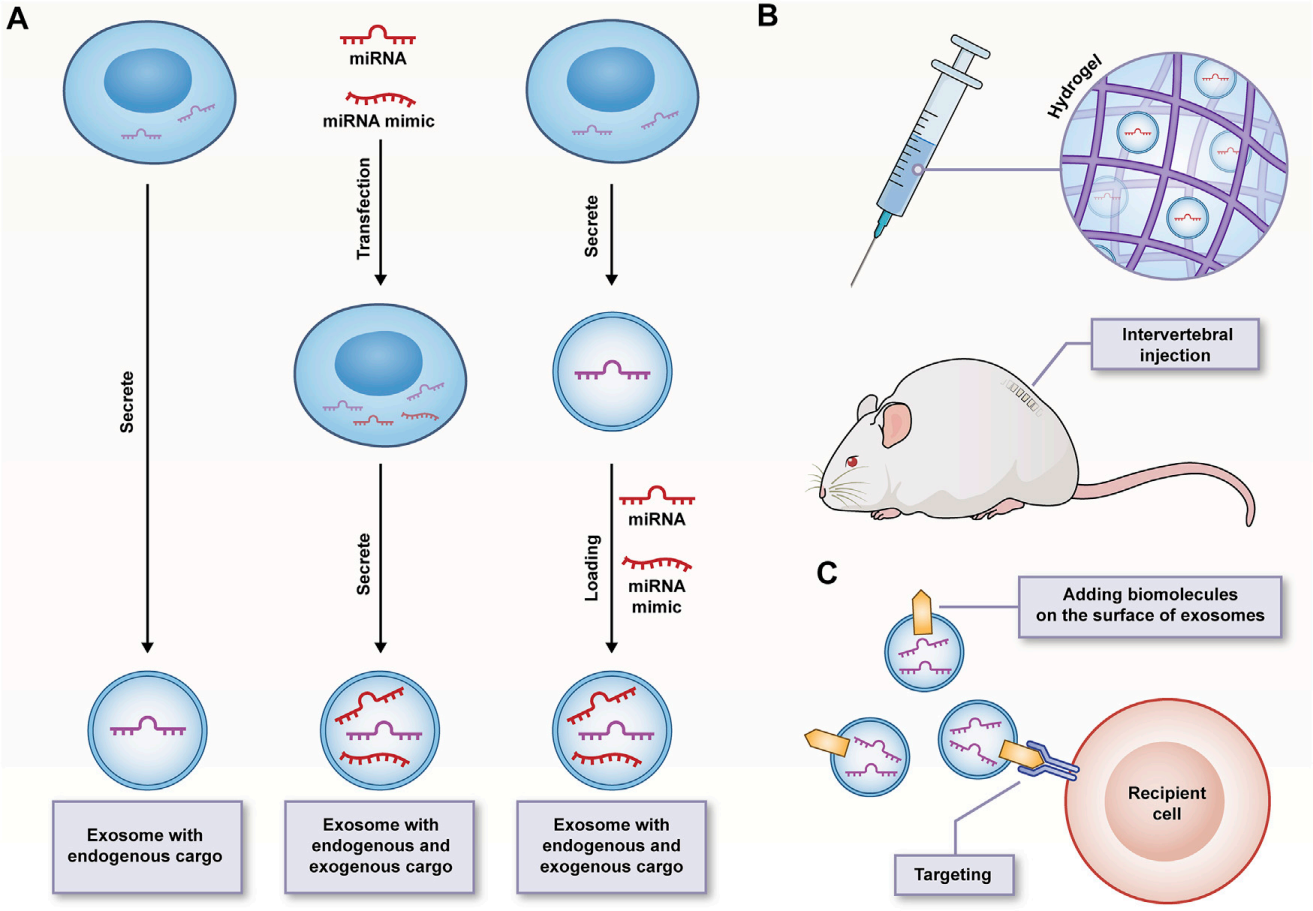
**(A)** Strategies of exosomes as transport vehicles for endogenous and exogenous miRNA/miRNA mimics; **(B)** The sustained-release properties and good biocompatibility of hydrogels facilitating safe delivery and durable function of exosomes; **(C)** Surface-functionalized exosomes improving targeting specificity.

Not only are intracellular miRNAs vital regulators of gene expression, the transfer of miRNAs through exosomes also plays an essential role in IVDD. Accordingly, over the past few years, exosomes and exosomal miRNAs have become the research focus in IVDD treatment. The existing research results have been summarized and classified in accordance with the different focus mechanisms of various studies, and the focus is placed on the regulatory roles of exosomes and exosomal miRNAs in apoptosis, senescence, and proliferation of IVD, as well as metabolic homeostasis, oxidative stress, inflammation, and angiogenesis of ECM (Table 1; Figure 4).

**TABLE 1:** The roles of exosomal miRNAs in IVDD.

Donor cells	miRNAs	Pathway	Function	References
MSCs	miR-21	PTEN/PI3K/Akt	Inhibits TNF-α-induced apoptosis of NPCs	Cheng X et al. (2018)
MSCs	miR-194-5p	TRAF6	Inhibits TNF-α-induced apoptosis of NPCs	Sun et al. (2022)
MSCs	miR-142-3p	MLK3/MAPK	Inhibits IL-1β-induced apoptosis of NPCs	Zhu G et al. (2020)
BMSCs	miR-155	BACH1, HO-1	Promotes autophagy in NPCs under glucose and oxygen deprivation and inhibits apoptosis	Shi et al. (2021)
CESCs	miR-125-5p	SUV39H1	Promotes TBHP-induced autophagy and inhibits apoptosis in NPCs	Chen and Jiang (2022)
BMSCs	miR-199a	GREM1/TGF-β	Delays the aging of NPCs and inhibits apoptosis	Wen et al. (2021)
iPSCs-MSCs	miR-105-5p	Sirt6	Delays the aging of NPCs	Sun Y et al. (2021)
BMSCs	miR-532-5p	RASSF5	Inhibits the apoptosis of NPCs induced by TNF-α, the degradation of ECM, the deposition of fibrosis, and restores the metabolic balance of ECM	Zhu L et al. (2020)
PRP	miR-141-3p	Keap1/Nrf2	Inhibits H2O2-induced cytotoxicity of NPCs	Xu et al. (2021)
MSCs	miR-31-5p	ATF6	Inhibit ER stress, inhibit apoptosis, and calcification of CEPCs induced by TBHP	Xie et al. (2020)
BMSCs	miR-129-5p	LRG1/p38 MAPK	Inhibits/Promotes M1/M2 polarization of macrophages	Cui and Zhang, (2021)
MSCs	miR-410	NLRP3/caspase-1	Inhibits LPS-induced pyroptosis of NPCs	Zhang et al. (2020)
hucMSCs	miR-26a-5p	METTL14	Inhibits pyroptosis	Yuan et al. (2021)
NCs	miR-140-5p	Wnt/β-catenin	Inhibits angiogenesis and IVD vascularization of endothelial cells	Sun et al. (2020b)
D-NPSCs	let-7b-5p	IGF1R/PI3K/Akt	Inhibits proliferation, migration, and matrix synthesis of AFCs, and promotes apoptosis	Zhuang et al. (2021)
TNF-α-NPCs	miR-16	IGF-1/IGF-1R	Promotes apoptosis of NPCs	Zhang et al. (2021a)
RAP-NPCs	miR-27a	MMP-13	Inhibits ECM degradation induced by IL-1β	Zhang et al. (2021b)
Hypoxic-MSCs	miR-17-5p	TLR4, PI3K/AKT	Promotes the proliferation of NPCs and the synthesis of ECM, and inhibits the apoptosis of NPCs and the degradation of ECM	Zhou Z. M et al. (2022)

### Apoptosis, senescence and proliferation

The number and activity of cells are the key to maintain the healthy state of IVD, and the cell death of IVD caused by the over activation of apoptosis program is an important cause of IVDD. Accordingly, in the regeneration process of IVD, the recovery of cell numbers and the delay of aging are the most important issues (Zhang X. B et al., 2021). In models of IVDD induced by conditions such as TNF-ɑ, IL-1β, tert-butyl hydroperoxide (TBHP), and glucose-oxygen deprivation, exosomes and exosomal miRNAs play the role of pro-proliferation and anti-apoptosis through different ways. For example, in a 3D *in vitro* model, treatment with exosomes derived from mesenchymal stem cells (MSCs) significantly enhanced the viability and proliferation of degenerative IVD cells obtained from patients with degenerative IVD and chronic low back pain, and inhibited their apoptosis (Hingert et al., 2020). Furthermore, there are a considerable number of miR-21 and miR-194-5p in MSCs-derived exosomes (MSCs-exos), thus inhibiting apoptosis of NPCs induced by TNF-α by activating the PTEN/PI3K/Akt pathway and targeting TRAF6, respectively (Cheng X et al., 2018; Sun et al., 2022). Similarly, MSCs-exos inhibited MAPK pathway transduction and IL-1β-induced apoptosis of NPCs by delivering miR-142-3p that targets MLK3 (Zhu G et al., 2020). Furthermore, exosomes derived from human placental mesenchymal stem cells (HPMSCs) carrying AntagomiR-4450 can significantly reduce the level of miR-4450 in TNF-α-induced mice *in vivo* and *in vitro*. By upregulating ZNF121, it can facilitate the proliferation and migration of NPCs, inhibit apoptosis and improve gait disorder. More importantly, this paper confirms that exosomes, as viable natural nanocarriers, show unique advantages in treating IDD by delivering drug molecules (e.g., antagomiR-4450), which are potential options for treating IDD (Yuan et al., 2020).

Autophagy refers to a stress-responsive intracellular survival mechanism. Activation of autophagy can properly correct injury or cellular stress. If the injury or cellular stress is too great for autophagy to overcome, it may cause apoptosis. Existing studies have found the presence of markers of autophagy in IVD tissues (Kritschil et al., 2022). A report suggested that bone marrow mesenchymal stem cells (BMSCs)-derived exosomes (BMSCs-exos) could inhibit autophagy by activating PI3K/AKT/mTOR signaling pathway, thus inhibiting the inflammation and apoptosis of IL-1β-induced AF cells (AFCs) and promoting the proliferation of AFCs (Li Z. Q et al., 2020). In contrast, another study found that BMSCs-exos-enriched miR-155 induced autophagy in NPCs under glucose and oxygen deprivation by facilitating the degradation of BACH1 and upregulating the expression of HO-1 protein; as a result, the apoptosis is inhibited (Shi et al., 2021). By regulating the AKT pathway or targeting SUV39H1 through its enriched miR-125-5p, exosomes derived from cartilage endplate stem cells (CESCs) can facilitate autophagy and inhibit TBHP-induced apoptosis in NPCs (Luo et al., 2021b; Chen and Jiang, 2022). The above autocrine exosomes can also facilitate their own migration and transdifferentiation into NPCs by activating the hypoxia-inducible factor (HIF)-1α/Wnt pathway and upregulating the expression of GATA4/TGF-β; as a result, IVDD is blocked (Luo et al., 2021a). It is therefore revealed that some research results on autophagy are contradictory, which seems to arise from the interference of different factors. These factors consist of the type of stimulation, the type of cells, and the source of exosomes, thus making it difficult to compare their results and conclusions. However, it is not difficult to judge that exosomes and exosomal miRNAs have a therapeutic effect on IVDD by regulating autophagy. Accordingly, subsequent standardized methods and model studies are worth looking forward to. Similar to autophagy, cells activate an adaptive response called unfolded protein response (UPR) in response to endoplasmic reticulum (ER) stress. However, the UPR is switched from an adaptive UPR to a pro-apoptotic UPR under continuous stress conditions of ER to trigger a pro-apoptotic program to eliminate existing cells, which leads to aggravation of the disease (Wu and Sun, 2021). It is reported that human urinary stem cells (USCS)—derived exosomes (USCS exos) significantly ameliorate ER stress and inhibit hyperactivation of UPR *via* AKT and ERK signaling pathways, and thereby stress-induced apoptosis in NPCs are inhibited (Xiang et al., 2020). Likewise, BMSCs-exos is capable of reducing apoptosis induced by ER stress by activating AKT and ERK signaling pathways in IDD related to end products of advanced glycosylation (Liao et al., 2019).

After chronic and long-term replication, cellular senescence occurs in the aging process of IVD as a natural link, whereas it is also promoted by factors (e.g., growth factor deficiency, oxidative accumulation, as well as inflammatory stimulation). Excessive senescence prevents cell cycle transition and stops proliferation, reduces cell viability and deteriorates the microenvironment, which is causally related to the occurrence of IVDD (Wang et al., 2016). Exosomal miR-199a and miR-105-5p are shown to be beneficial in delaying the senescence of IVD cells. To be specific, miR-199a is enriched in BMSCs-exos, and it can inactivate the TGF-β pathway by targeting GREM1, resulted in a significant decrease in the number of cells and apoptosis of senescence-associated β-galactosidase (SA-βgal) positive NPCs and promoted the proliferation of NPCs (Wen et al., 2021). Exosomes derived from MSCs derived from induced pluripotent stem cells (iPSCs) are capable of delivering miR-105-5p to aging NPCs and activating the Sirt6 pathway, and the aging phenotype and age-related dysfunction of NPCs are ameliorated (Sun Y et al., 2021) (Table 1).

### Homeostasis of the extracellular matrix

Metabolic disturbance of the ECM is the most striking feature of IVDD. Thus, in studies on IVDD, the enhancement of anabolism should be emphasized, and catabolism should be reduced to restore ECM homeostasis, which is of critical significance to maintain ECM integrity and slow IVDD. Recent evidence demonstrates that exosomes and exosomal miRNAs are capable of regulating the gene expression level of synthesis and catabolism of ECM and effectively storing ECM homeostasis. It has been reported that after treating IVD cells from IVDD patients with MSCs-exos for 7 days, ECM production was observed to be more than three-fold higher than that of control group, and MMP-1 expression was significantly inhibited (Hingert et al., 2020). Likewise, NPCs and BMSCs have also been observed to complete information exchange through exosomes. NPCs-derived exosomes promoted the migration of BMSCs and induced the differentiation of BMSCs into nucleus pulposus cells, while BMSCs-exos promoted the proliferation of degenerated NPCs and the production of ECM (Lu et al., 2017). Besides, BMSCs-exos can downregulate the expression of MMP-13 and upregulate the expression of aggrecan and type II collagen in pathological acidic environments. Moreover, its enriched miR-532-5p can target RASSF5 to inhibit TNF-α-induced apoptosis, ECM degradation and fibrotic deposition in NPCs, and the balance of synthesis and catabolism of ECM can be restored (Li M et al., 2020; Zhu G et al., 2020). Furthermore, USCs-exos may serve as a potentially effective drug to alleviate IVDD by transferring MATN3 protein, so as to facilitate NPCs proliferation and ECM synthesis, while MATN3 was found to be effective in protecting human NPCs from ECM degeneration in previous studies, which was correlated with the amount of collagen type II and aggrecan it maintains, as well as inflammation suppression (Lu et al., 2020; Guo Z et al., 2021) (Table 1).

### Oxidative stress

A wide range of adverse microenvironments can result in intracellular mitochondrial dysfunction and excessive levels of mitochondrial ROS in IVD, and antioxidative strategies such as antioxidant administration and repair of mitochondrial dysfunction may benefit the treatment of IVDD (Zhang C et al., 2022). It is observed that BMSCs-exos could significantly reduce ROS and MDA levels, inhibit stress-induced oxidative stress of NPCs, increase mitochondrial membrane potential, reduce mitochondrial damage, and promote the proliferation and activity of NPCs (Hu et al., 2021). H2O2 and TBHP can be employed to design oxidative stress conditions. At concentrations between 5–50 mg/ml, lyophilized MSCs-exos can counteract H2O2-induced oxidative stress injury in NPCs (Bari et al., 2018). Platelet-rich plasma (PRP)-derived exosomes, enriched with miR-141-3p, ameliorate the cytotoxic effect of H2O2 on NPCs by activating the Keap1/Nrf2 pathway and inhibit the progression of IVDD in mice (Xu et al., 2021). Furthermore, MSCs-exos are enriched in miR-31-5p, thus targeting and inhibiting ATF6-related ER stress, so apoptosis and calcification of TBHP-induced CEP cells are inhibited. Furthermore, subendplate injection of exosomes can significantly increase CEP thickness and maintain intact structure in IVDD rats (Xie et al., 2020).

Increasing evidence demonstrates that the role of mitochondria also extends to intercellular communication. This intercellular translocation ability that can involve the whole mitochondria, mitochondrial genome or other mitochondrial components can provide exogenous mitochondrial sources to supplement dysfunctional mitochondria, so as to reduce mitochondrial defects. More interestingly, exosomes take on a critical significance in mitochondrial transfer (Singh et al., 2017; Valenti et al., 2021). MSCs-exos can provide mitochondria-related proteins to NPCs, effectively restore mitochondrial dysfunction, and in the process inhibit the production of ROS and the activation of the inflammasome of NLRP3, and the apoptosis of NPCs is inhibited. *In vivo*, exosomes are capable of protecting the content of proteoglycan in IDD rabbit model, inhibiting ECM degradation, and slowing IVD progression (Xia et al., 2019) (Table 1).

### Inflammatory reaction

Stimulated by different causes, the IVDD continues to progress along with a chain of structural destruction events. However, inflammation has been always seen regardless of the cause or stage of IVDD progression (Molinos et al., 2015). Degenerated IVD tissues can spontaneously produce chemokines (e.g., IL-8 and MMP-1), whose main functions are chemotaxis of macrophages and induction of angiogenesis (Burke et al., 2002). By inhibiting LRG1 expression, BMSCs-exos carrying miR-129-5p attenuates its mediated activation of the p38 MAPK pathway to prevent M1 polarization of macrophages, while facilitating M2 polarization to release anti-inflammatory factors, and ultimately prevent NPCs Apoptosis and ECM degradation (Cui and Zhang, 2021). The inflammasome has been found as a vital component of innate immunity. NLRP3, as a representative member, is sensitive to various inflammatory-induced stimuli. Recent studies have demonstrated that it is widely activated during IVDD (Chao-Yang et al., 2021). miR-410 in MSCs-exos can inhibit LPS-induced pyroptosis in NPCs by inhibiting the NLRP3/caspase-1 pathway and reversing the expression levels of IL-18 and IL-1β (Zhang et al., 2020). Likewise, miR-26a-5p, enriched in human umbilical cord mesenchymal stem cell (hucMSCs)-derived exosomes, targets the mRNA-methyltransferase (METTL14) in NPCs. It reduces the level of NLRP3 in NPCs by affecting m6A methylation, and results in reduced proinflammatory cytokine release and pyroptosis (Yuan et al., 2021) (Table 1).

### Angiogenesis

AF structure is a natural physical barrier for IVD to block the inward growth of blood vessels. It is destructed by the release of pro-angiogenic factors and pro-inflammatory cytokines, thus inducing vascular invasion, exposing NPs to the immune system, and ultimately leading to the complete destruction of the internal environment of IVD (Li et al., 2021). As revealed by an earlier study, “microparticles” and soluble protein factors secreted by endothelial cells were taken up by AFCs and disrupted the homeostasis of the inner matrix in IVD (Pohl et al., 2016). As reported by a recent study, exosomes derived from degenerated AFCs can be absorbed by endothelial cells, thus facilitating their migration and the expression of inflammatory factors and promoting angiogenesis. While exosomes derived from non-degenerated AF can be active inhibitors to inhibit angiogenesis (Sun Z et al., 2021). The above two studies confirmed that exosomes are novel mediators of intercellular communication in neovascularization of IVDD, which suggest that early intervention of exosomes in degenerative AFCs should be implemented. The disappearance of NCs often marks the onset of early IDD, so people are always interested in the underlying mechanisms by which NCs maintain the healthy state of IVD and see the above mechanisms as hope for IVD regeneration. To date, therapeutic NCs have been found to promote matrix production of NPCs, increase cell proliferation, reduce apoptosis, and inhibit angiogenesis and neurogenesis (Bach et al., 2021). A recently study has also confirmed the therapeutic effect of NCs-derived exosomes. The above exosomes were rich in miR-140-5p cultured under a compressive load of 0.5MPa, which inhibited angiogenesis of endothelial cells *via* the Wnt/β-catenin pathway and significantly reduced vascularization in degenerated IVD tissues of mice *in vivo* (Sun et al., 2020b) (Table 1).

### Pretreatment of exosomes

Similar to the droplets from a cold patient’s sneeze, exosomes can also reveal the state of the cell of origin, and there is great interest in the role of exosomes derived from IVD cells with an abnormal degenerative phenotype. Several studies have assessed the effect of exosomes on downstream responses in the physio-pathological state. There are a considerable number of let-7b-5p in exosomes derived from degenerative IVD nucleus pulposus stem cells (D-NPSCs), which can target to inhibit the activation of IGF1R/PI3K/Akt pathway for inhibiting the proliferation, migration and matrix synthesis of AFCs and facilitating the apoptosis of AFCs (Zhuang et al., 2021). Furthermore, exosomes derived from TNF-α-induced degenerated NPCs could induce the apoptosis of CEPCs and aggravate the degeneration of IVD in rats. Besides, their enriched exosomal miR-16 could directly inhibit the anti-apoptotic IGF-1/IGF-1R pathway to facilitate the apoptosis of normal NPCs (Zhang et al., 2021a; Feng et al., 2022). Furthermore, exosomes derived from NPCs with a senescent phenotype induced by IL-1β can accelerate the senescence of normal NPCs by activating the P53/P21 pathway (Chen et al., 2021).

Since the exosomes derived from IVD cells with abnormal degenerative phenotype can promote the progress of IVDD, a question is raised that whether we can make IVD cells release stronger exosomes through pretreatment. It seems feasible. NPCs treated with rapamycin (RAP) derived more exosomes and increased the expression of mir-27a in exosomes, which attenuate IL-1β-induced ECM degradation in NPCs by targeting MMP-13 (Zhang et al., 2021b). Likewise, hypoxic preconditioning seems to be more favorable for the generation of BMSCs-derived exosomes. More interestingly, the above exosomes are more easily taken up by NPCs. It targets the inhibition of TLR4 activity and activates the PI3K/AKT pathway by delivering abundant miR-17-5p, and promotes the proliferation of NPCs and inhibits their apoptosis. Upregulated expression of aggrecan and collagen II and downregulated expression of MMP-13 and ADAMTS-5 promote expression of ECM components and reduce matrix degradation (Zhou Z. M et al., 2022) (Table 1).

## Discussion

The research based on miRNAs has been gradually shifting to the stage of clinical experiment. However, a drug delivery system with stronger stability, lower clearance rate, better biocompatibility and immune tolerance is urgently required, and more specific targeting effect. With the identification of exosomes as intercellular messengers, there has been intense interest in the study of exosomes. They are capable of carrying a wide variety of goods shuttle in cells, while simultaneously transmitting different signals to achieve real-time intercellular communication. The nanoscale size and special structure of exosomes make it easier for them to escape the phagocytosis of lysosomes, their contents are better protected, and they can support the distance of systemic transport. Self-derived exosomes are less immunogenic, and the presence of a hydrophilic center makes them easier to encapsulate water-soluble drugs (Wahlgren et al., 2012; Ha et al., 2016; Jiang and Gao, 2017; Marbán, 2018). On that basis, exosomes are indicated as a perfect fit for the delivery system required for miRNAs therapy. Thus, therapeutic researches based on exosomes and exosomal miRNAs have been extensively conducted in various disease fields. IVDD is also among them, and promising results relating to IVDD have been achieved.

However, the potential and advantages of this emerging treatment strategy are not significant since there are numerous problems to be solved. Combined with the existing research progress, whether we can effectively restore the number and density of cells in IVD is dependent on the stability of apoptosis, cell aging and proliferation. Likewise, most studies have been conducted. These studies have emphasized the role of exosomes and exosomal miRNAs in intervening the apoptosis, aging and proliferation of NPCs, but rarely involve AFCs (only 3 items) and CEPCs (only 3 items). It is necessary to gain a full insight into the regeneration and protective characteristics of this method on all terminal differentiation types of cells in IVD. The change of miRNAs expression profile is significantly correlated with IVDD, thus providing an opportunity for exosomal miRNAs as therapeutic targets to counteract the degradation process. Moreover, to improve the therapeutic effect of exosomal miRNAs, target miRNAs or miRNA mimics can also be directly transfected into exosomes through electroporation, ultrasound and freeze-thaw cycle (Kim et al., 2020) (Figure 4A). However, the above complex factor network is highly difficult to be regulated through a few exosomal miRNAs. It may be necessary to screen more highly selective exosomal miRNAs that can participate in all aspects of IVDD or play a leading role in different stages of IVDD. In addition, the wider miRNAs-mRNAs network also needs to be expanded. The recruitment of cells from the surrounding environment to replenish itself is considered a vital aspect of the IVD regeneration process, and NPCs-exos have well demonstrated their chemotaxis to MSCs (Lan et al., 2019), which may make a good supplement to the cell viability and healthy matrix required in IVD. However, further *in vivo* experiments should be performed to demonstrate whether its induction efficiency is affected by the content of exosomes and the type and state of originating cells, whether the complex environment in degenerated IVD will interfere with the migration rate of recruited cells, and whether the cytokine secretion of activated MSCs will also be affected.

The avascular nature within IVD has an effect on the choice of administration route of exosomes. Assuming that intervertebral injection is an ideal drug delivery method, it will face the problem of short action time and fails to meet the long-term curative effect and the need for repeated injections. Accordingly, the half-life of exosomes should be extended *in vivo*, and their biologically active functions should be maintained. The researchers built a system of thermosensitive cell-free ECM hydrogel combined with ADSCs-exos, capable of significantly maintaining the microenvironment homeostasis in early IVD and inhibiting pyroptosis of NPCs (Xing et al., 2021). The slow-release properties and better biocompatibility of ECM hydrogel may contribute to the safe delivery and long-term functional development of the exosomes (Figure 4B). The systemic administration of MSCs-exos through caudal vein injection was also found with a certain therapeutic effect. Thus, it is challenging to improve the targeting specificity of exosomes for shortening the targeting time. The development of surface-functionalized exosomes may be promising to solve the above problem, which has been conducted in some diseases (Cheng Q et al., 2018; Zhang J et al., 2022). However, there has been rare relevant report in the field of IVDD. Regardless, the engineering of exosomes appears to be an essential step before clinical application (Figure 4C).

## Challenges and future perspectives

Exosomes and exosomal miRNAs show a wide range of promise in the field of IVDD therapy.They have been confirmed to prevent or even reverse the process of IVDD by regulating IVD cell apoptosis, restoring ECM homeostasis, reducing inflammatory response, antagonizing oxidative stress injury, and inhibiting angiogenesis. However, despite the recognized therapeutic potential, fundamental issues of exosomes and exosomal miRNAs for the treatment of IVDD remain, such as efficient and pure exosome production methods and standards, comprehensive IVD cell targeting studies, reasonable dosing and frequency criteria for administration, appropriate vector selection or pretreatment, accurate and sensitive differential expression analysis of miRNAs, and efficacy assessment of methods and criteria, all of which need to be confirmed by subsequent studies. Additionally, the fact that exosomes are cell-derived but not cellular may pose challenges to their legal classification and obtaining regulatory approval for use in clinical trials in various countries (Liao et al., 2018). For now, only one group is conducting a clinical trial of exosome therapy for IVDD (https://clinicaltrials.gov/ct2/show/NCT04849429), consisting of intradiscal injection of exosome-rich autologous platelet-rich plasma in 30 participants, scheduled completion by 25 July 2022, however, no specific experimental results have been reported yet, and it is expected that this clinical trial can provide a reference for subsequent clinical trials to advance. In brief, as a novel strategy for treating IVDD, exosomes and exosomal miRNAs bring both hope and challenges. However, we consider that the solution of the above basic and clinical problems will bring great surprises to the reversal of IVDD.
